# Supplementary material for: A Novel PDMS-Based Flexible Thermoelectric Generator Fabricated by Ag2Se and PEDOT:PSS/Multi-Walled Carbon Nanotubes with High Output Performance Optimized by Embedded Eutectic Gallium–Indium Electrodes
Source: Nanomaterials (Basel). 2024 Mar 20;14(6):542. doi: 10.3390/nano14060542 (PMC10975368; doi:10.3390/nano14060542)
Supplement: Supplementary file 1 [file nanomaterials-14-00542-s001.zip › nanomaterials-2899203-supplementary.pdf]

A Novel PDMS-based Flexible Thermoelectric Generator Fabricated by Ag2Se and PEDOT:PSS/multi-walled carbon nanotubes with High Output Performance Optimized by Embedded Eutectic Gallium–Indium Electrodes

Rui Guo[a], Weipeng Shi[a], Rui Guo[a], Chenyu Yang[a], Yi Chen[a], Yonghua Wang[a], Danfeng Cui[a], Dan Liu[a]*, Chenyang Xue[a]

a. State Key Laboratory of Dynamic Measurement Technology, North University of China, Taiyuan 030051, China

* Correspondence author: Dan Liu (liudan235@nuc.edu.com)

Section S1

Experimental Section

1.1 Fabrication of thermoelectric columns

Firstly, using a pipette put 1g of PEDOT: PSS into a beaker and measure a volume ratio of 5% DMSO into the beaker. The mixed solution was mixed well with the ultrasonic shaker. A certain amount of MWCNT was added to the mixed solution and stirred evenly. The mass fractions of MWCNTs were 5wt%, 10wt%, 15wt%, and 20wt%, respectively. Using a rubber-tipped dropper, drop the mixed solution into a cold press mold, heat on the hot table for 5 minutes, and the mixed solution becomes gel-like; repeat the process until the mixed solution is used up. Thermoelectric columns were prepared at the same pressure and named PM5, PM10, PM15, and PM20, respectively. Meanwhile, the PEDOT: PSS thermoelectric column without MWCNTs was named PM0. After conducting the thermoelectric property tests, the best mass ratio was selected for subsequent experiments. The cold pressing fabrication was taken at 0, 2, 4, 6, 8, 10, and 12 MPa and named as P0, P2, P4, P6, P8, P10, and P12, respectively.

For the Ag_2Se thermoelectric column, our group did a related study on Ag_2Se in the previous stage. According to the research, the weight ratio of Ag_2Se to PVP was chosen as 30:1 to prepare the thermoelectric column, and the fabrication process was the same as that of the P-type thermoelectric column. In the fabrication process, 0, 2, 4, 6, 8, 10, and 12 MPa were taken to prepare Ag_2Se thermoelectric columns named N0, N2, N4, N6, N8, N10, and N12. All n/p type thermoelectric columns are cylinder with a height and diameter of 3mm in Fig S1.

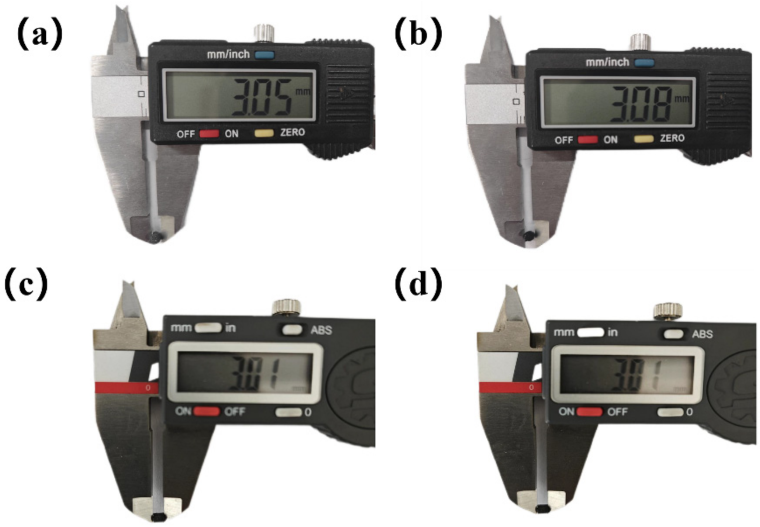

Figure S1. The size of the thermoelectric column is measured by (a) the height of the n-type thermoelectric column, (b) the p-type thermoelectric column, the diameter of (c) the n-type thermoelectric column, and (d) the p-type thermoelectric column.

1.2 Performance characterization of the thermoelectric columns

The thermoelectric column was tested for output voltage and current. The test results are shown in Fig S2. Fig S2 shows that the σ of the mixtures increased with the increase in the content of MWCNTs. MWCNTs have excellent electrical conductivity, and the increase in their content increases the conductive channels in the mixture. Moreover, the increase in the content of MWCNTs increases the mutual contact between MWCNTs and PEDOT.PSS, thus increasing the conductivity of the mixture. On the other hand, Fig S2 shows that the *S* of the mixture is maximum at 10% of MWCNTs, which is 21.71 μV/K. There is an energy filtering effect due to the PEDOT:PSS-MWCNTs junctions[1]. When the content of MWCNTs is low, the energy barrier hinders the transport of low-energy carriers. And when the content of MWCNTs increases further, the energy filtering effect disappears due to the interconnection of CNTs. Therefore, the *S* increases and then decreases with increasing of MWCNTs. As shown in Fig S2, the nonlinear variation of the *S* of the mixture leads to the maximum power factor (*PF*) at a 10% weight ratio of MWCNTs, which is 9.25 $\mu W\cdot m^{-1}\cdot K^{-2}$, so the mixture of 10wt% MWCNTs was chosen for the subsequent experiments. The conductivity of the thermoelectric column was measured with a four-probe semiconductor analyzer.

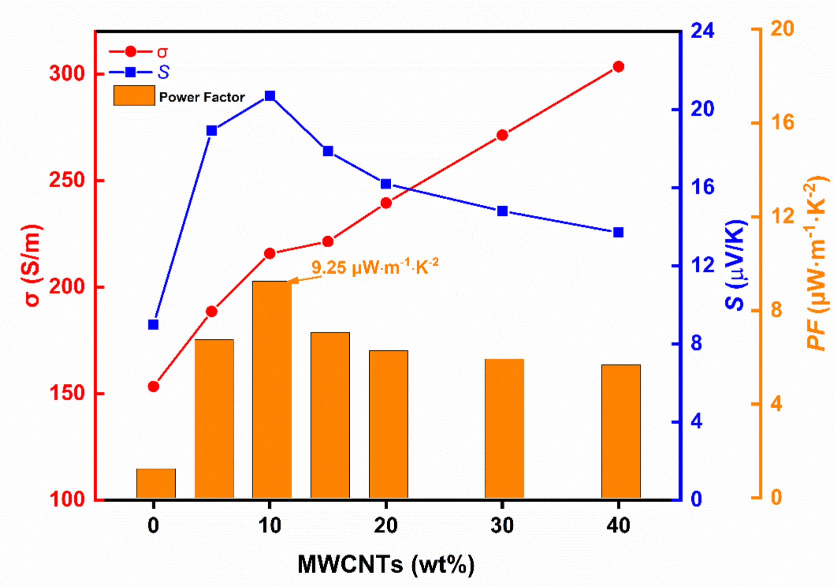

Figure S2 The effect of different MWCNTs content on the conductivity(σ), Seebeck coefficient(S), and power factor(PF) of the thermoelectric column.

The S of n-type and p-type columns are shown in Fig S3. Fig S3a and 3c show that the S of n-type columns gradually increases with increasing pressure, but decreases at 12 MPa. S is maximum at 10 MPa with 43.9 μV/K. Different fabrication pressures caused a small shift of atomic positions in Ag_2Se, which led to an increase in the density of defects in the lattice and an increase in the scattering, causing the S of Ag_2Se to increase with the increase in fabrication pressure. For the p-type thermoelectric columns, the variation of S with pressure is not obvious about 21 μV/K in Fig 3b and 3c. Localized strain under pressure leads to increased phonon-disordered motion and phonon scattering. However, MWCNTs have high thermal conductivity, and the high pressure makes the MWCNTs more tightly packed, the phonon concentration increases, and the thermal conductivity of the thermoelectric columns increases, thus making the thermoelectric output of the p-thermoelectric columns show a disordered variation, resulting in a small change of S.

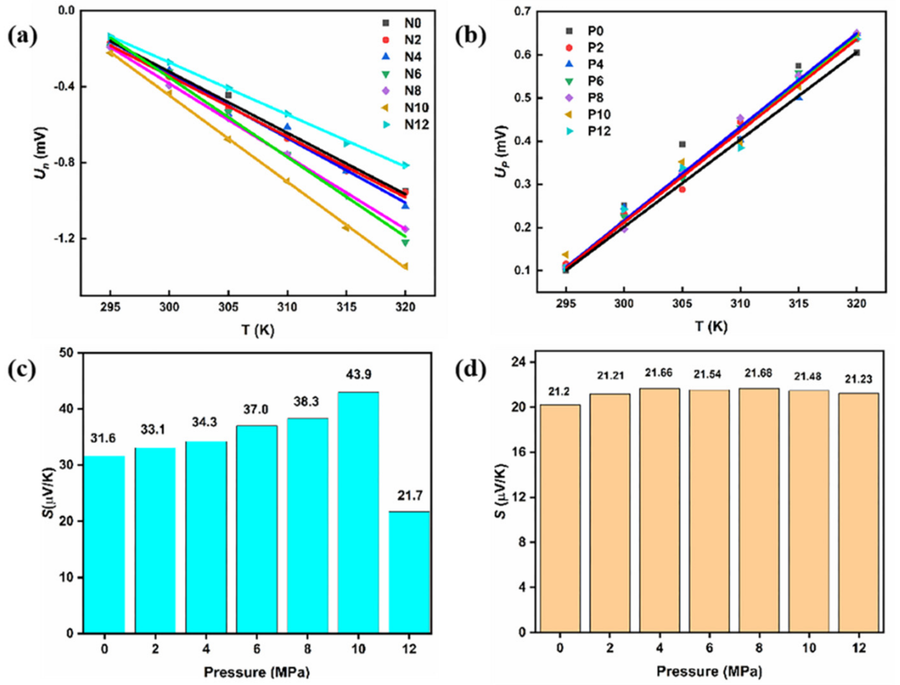

Figure S3 (a) Ag_2Se output voltage versus temperature at different fabrication pressures, (b) PEDOT:PSS/MWCNTs' output voltage versus temperature at different fabrication pressures, (c) effect of fabrication pressure on the *S* of Ag_2Se, and (d) effect of fabrication pressure on the *S* of PEDOT:PSS/MWCNTs.

The conductivity test results of the thermoelectric column are shown in Table S1. All n-type and p-type thermoelectric columns exhibit the same trend of change. Increasing pressure can lead to tighter packing of the material in the thermoelectric column. First, the tighter packing of the material can increase the carrier concentration. More material is packed into the same volume, which means there are more carriers available for conduction. This increased carrier concentration can result in an increase in electrical conductivity. Second, the reduction in the presence of air or voids between the materials in the thermoelectric column can decrease the internal resistance of the material. Air or voids can act as barriers to electron flow, increasing the resistance. By reducing these barriers, the internal resistance of the material can decrease, leading to better electrical conductivity.

Table S1 Effect of different fabrication pressures on the conductivity of n/p thermoelectric columns

Pressure (MPa)	σ_p(S/m)	σ_n(S/m)
0	215.7	49.33
2	218.2	53.13
4	220.4	59.31
6	220.8	65,44
8	222.1	71.45
10	224.8	78.33
12	225.3	85.64

1. J.-H. Hsu, W. Choi, G. Yang and C. Yu, *Organic Electronics*, 2017, **45**, 182-189, https://doi.org/10.1016/j.orgel.2017.03.007.

Section S2

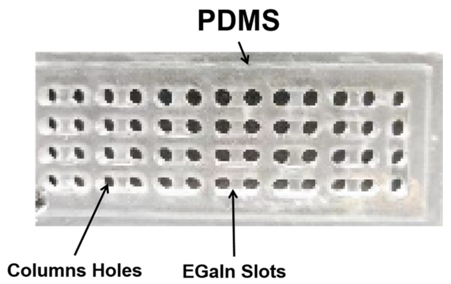

Figure S4. Schematic diagram of the PDMS framework.

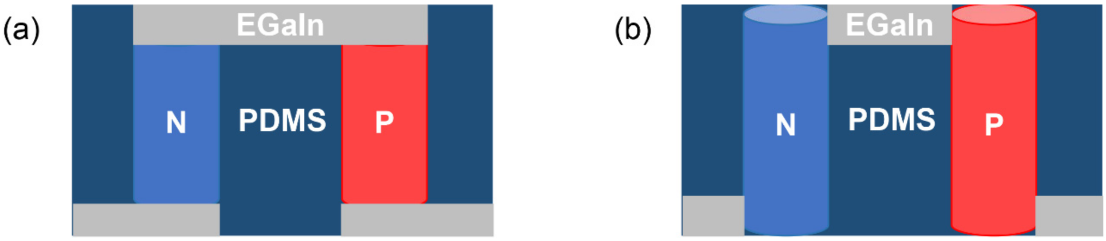

Figure S5. Schematic diagram of different electrodes.

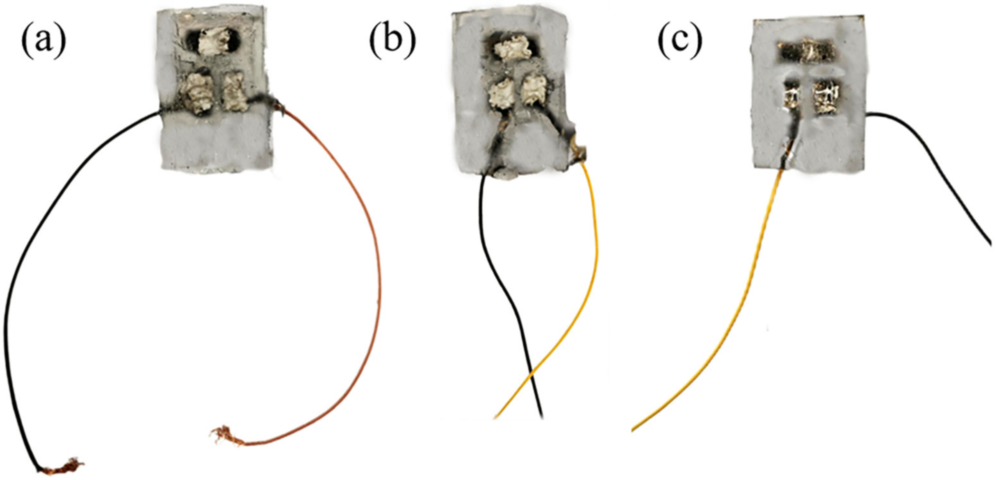

Figure S6 (a) The physical picture of t-FTEG, (b) l-FTEG, and (c) nl-FTEG

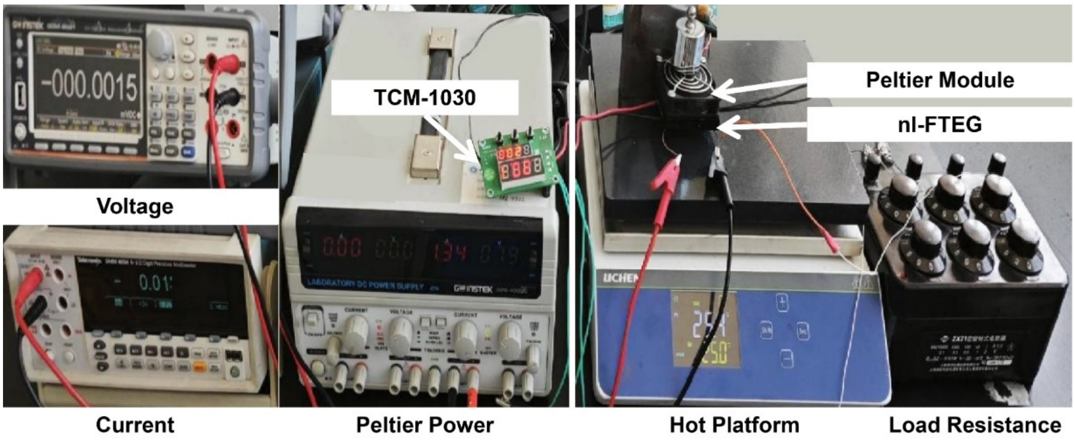

Figure S7. The test system of the output voltage and output power of the FTEG

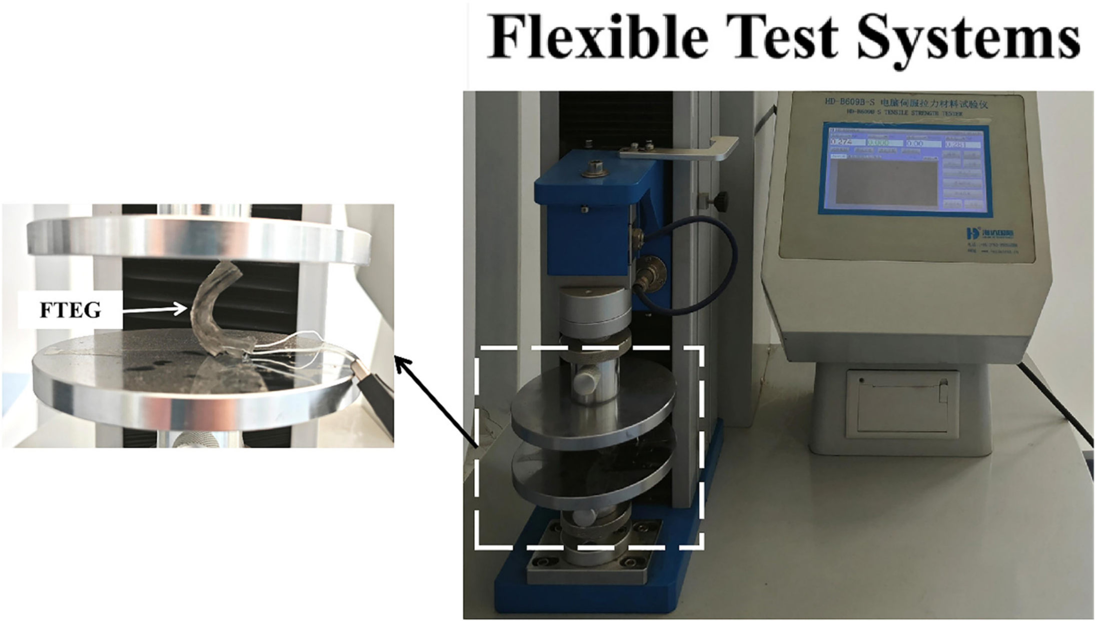

Figure S8 Actual test chart of FTEG flexibility
